# Robotic‐assisted total knee arthroplasty reduces postoperative complications and length of stay without increased cost compared to navigation‐guided techniques: A national analysis

**DOI:** 10.1002/ksa.12348

**Published:** 2024-07-02

**Authors:** David Maman, Lior Laver, Roland Becker, Assil Mahamid, Yaron Berkovich

**Affiliations:** ^1^ Department of Orthopedics Carmel Medical Center Haifa Israel; ^2^ Rappaport Faculty of Medicine Technion University Hospital (Israel Institute of Technology) Haifa Israel; ^3^ Department of Orthopedics Hillel Yaffe Medical Center Hadera Israel; ^4^ Department of Orthopedics and Traumatology University Hospital Brandenburg, Brandenburg an der Havel Berlin Germany

**Keywords:** NIS, postoperative complications, robotic surgery, robotic total knee arthroplasty, total knee arthroplasty

## Abstract

**Introduction:**

This study compares postoperative outcomes of robotic‐assisted total knee arthroplasty (RA‐TKA) versus navigation‐guided total knee arthroplasty (NG‐TKA). Using Nationwide Inpatient Sample (NIS) data, it provides an analysis of postoperative complications, mortality, hospital costs and duration of stay.

**Methods:**

The study analysed 217,715 patients (81,830 RA‐TKA; 135,885 NG‐TKA) using NIS data from 2016 to 2019. Elective TKA patients were identified through the International Classification of Diseases, 10th Revision codes. Statistical analyses, including logistic regression modelling, were performed using Statistical Package for the Social Sciences and MATLAB.

**Results:**

RA‐TKA patients were younger (66.1 vs. 67.1 years, *p* < 0.0001) and had similar mortality rates (0.024% vs. 0.018%, *p* = 0.342) but shorter length of stay (LOS) (1.89 vs. 2.1 days, *p* < 0.0001). Mean total charges were comparable between RA‐TKA ($66,180) and NG‐TKA ($66,251, *p* = 0.669). RA‐TKA demonstrated lower incidences of blood‐related complications (11.67% vs. 14.19%, *p* < 0.0001), pulmonary oedema (0.0306% vs. 0.066%, *p* < 0.0001), deep vein thrombosis (0.196% vs. 0.254%, *p* = 0.006) and acute kidney injury (AKI) (1.356% vs. 1.483%, *p* = 0.016).

**Conclusion:**

RA‐TKA reduces postoperative complications and LOS without increasing costs, highlighting the relevance of this technology in patient care.

**Level of Evidence:**

Level III.

**Abbreviations**
AKI: acute kidney injury
C‐TKA: conventional total knee arthroplasty
HCUP: Healthcare Cost and Utilisation Project
ICD‐10: International Classification of Diseases, 10th Revision
KSS: Knee Society Score
LOS: length of stay
NG‐TKA: Navigation‐Guided Total Knee Arthroplasty
NIS: Nationwide Inpatient Sample
RA‐TKA: robotic‐assisted total knee arthroplasty
SPSS: Statistical Package for the Social Sciences
SSI: surgical site infection
TKA: total knee arthroplasty
WOMAC: Western Ontario and McMaster Universities Osteoarthritis Index

## INTRODUCTION

The demand for total knee arthroplasty (TKA) has increased due to rising life expectancy and the high incidence of osteoarthritis. Two technologies, navigation‐guided total knee arthroplasty (NG‐TKA) and robotic‐assisted total knee arthroplasty (RA‐TKA), have been introduced to enhance surgical precision and outcomes [1, 2, 10, 19].

TKA incidence in the United States is the highest globally at 235 procedures per 100,000 individuals, with demand expected to nearly double by 2030 and triple by 2040 [7, 18, 25]. RA‐TKA's growing popularity stems from its potential to augment surgical accuracy, expedite recovery and enhance knee mobility [1, 4, 5, 7, 8, 15, 21, 23, 26]. NG‐TKA uses real‐time, three‐dimensional information to refine implant positioning and improve surgical precision [1, 4, 5, 7, 20, 23].

Despite previous studies, a critical gap remains in comparing RA‐TKA to NG‐TKA across a range of postoperative outcomes [1, 2, 3]. This study addresses this gap using a recent data set with a larger sample size of RA‐TKA procedures, providing a more robust and current analysis of these technologies. The analysis focuses on immediate and short‐term outcomes, including postoperative complications, hospital costs and length of stay (LOS). By leveraging this larger and more recent data set, our study provides new insights into the effectiveness of RA‐TKA, making it clinically relevant for decision‐making in surgical practices.

### Hypothesis

RA‐TKA decreases the risk of surgical complications and LOS due to increased accuracy and precision during the procedure.

## MATERIALS AND METHODS

### Data set acquisition and inclusion criteria

This study used a data set extracted from the Nationwide Inpatient Sample (NIS) database, the largest publicly available all‐payer inpatient care database in the United States. Each entry in the data set, referred to as a ‘case’, represented a group of five patients, resulting in a total of 217,715 patients from 43,543 cases. Amongst these cases, 81,830 involved RA‐TKA and 135,885 involved NG‐TKA. The NIS discharge weight indicates that each case extrapolates to five patients.

### Study period and data source

The data set spans from 1 January 2016 to 31 December 2019, representing the latest available information within the NIS system at the time of this study. The NIS is a crucial component of the Healthcare Cost and Utilisation Project (HCUP).

### Patient identification and exclusions

Patients undergoing TKA were identified using specific International Classification of Diseases, 10th Revision (ICD‐10) procedure codes related to elective total knee replacement. Exclusions included patients with nonelective admissions or revision surgeries.

### Statistical analyses

Statistical analyses, including logistic regression modelling, were conducted using Statistical Package for the Social Sciences (SPSS) 26 and MATLAB, with a *p* value threshold of less than 0.05 considered statistically significant. Comorbidities were identified and validated through a meticulous review of patient‐specific ICD‐10 codes. Cases with reported hospital costs as $0 were excluded. Analytical studies were performed to visualise annual cases, discern trends and derive key statistical insights. Microsoft Excel was utilised for data visualisation.

### Adjusting for confounders

To account for the potential confounding effects of pre‐existing chronic anaemia and chronic kidney disease (CKD) on postoperative outcomes, logistic regression modelling was employed. Both chronic anaemia and CKD diagnoses were incorporated as covariates within the model, enabling the isolation of the independent impact of the surgical system on patient outcomes.

### Outcome measures

Clinical outcomes, including in‐hospital mortality, LOS, in‐hospital complications and overall cost of hospitalisation, were explored for both RA‐TKA and NG‐TKA. Hospital costs were estimated by multiplying hospital charges by the given year's cost‐to‐charge ratios provided by HCUP. All statistical analyses were conducted using SPSS 26 and MATLAB, maintaining a *p* value threshold of less than 0.05 for statistical significance.

## RESULTS

**Figure 1:**
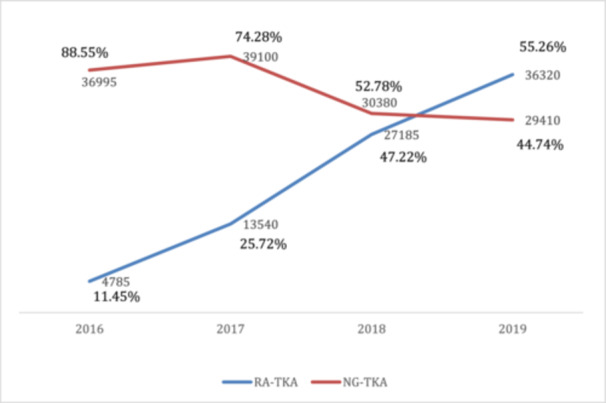
Annual trend of robotic‐assisted and navigation‐guided total knee arthroplasty (NG‐TKA) procedures (2016–2019). This figure illustrates the annual trend and proportionate distribution of robotic‐assisted total knee arthroplasty (RA‐TKA) and NG‐TKA procedures over 4‐year period from 2016 to 2019.

Figure 1 illustrates the absolute numbers and proportional distribution of RA‐TKA and NG‐TKA procedures from 2016 to 2019. The RA‐TKA procedures increased significantly, reaching 55% of the surgeries by 2019. The observed trends between the two methodologies and the variations in each calendar year are statistically significant, with a *p* value < 0.001.

To provide an understanding of the patient population undergoing RA‐TKA and NG‐TKA, we examined demographic parameters. Table 1 presents the demographic characteristics of patients undergoing RA‐TKA and NG‐TKA.

**Table 1:** Demographic characteristics of patients undergoing RA‐TKA and NG‐TKA.

Parameter	RA‐TKA	NG‐TKA	Significance
Total surgeries (%)	81,830	135,885	
Average age (years)	66.1	67.1	*p* < 0.0001
Female (%)	58	61	*p* < 0.0001
Race (%)			
White	83.7	81.8	*p* < 0.0001
Black	6.5	7.5	
Hispanic	5.4	6.5	
Asian or Pacific Islander	1.4	1.4	
Native American	0.9	0.8	
Other	2.1	2	
Payer (%)			
Medicare	54.4	57.9	*p* < 0.0001
Medicaid	3.2	3.5	
Private	38.2	34.9	
Other (including self‐pay)	4.2	3.7	
Median household income (%)			
0–25	20.8	21.5	*p* < 0.0001
26th–50th	25.1	28.1	
51th–75th	27.2	25.5	
76th–100th	26.9	24.9	

Abbreviations: NG‐TKA, navigation‐guided total knee arthroplasty; RA‐TKA, robotic‐assisted total knee arthroplasty.

Table 1 provides key demographic characteristics of patients undergoing RA‐TKA and NG‐TKA, including total surgeries, average age, gender distribution, racial composition, payer distribution and median household income.

In addition to demographic characteristics, we examined the prevalence of pre‐existing health conditions amongst patients undergoing RA‐TKA and NG‐TKA. Table 2 presents the percentages of patients with specific diagnoses, highlighting statistically significant differences between the two groups.

**Table 2:** Pre‐existing health conditions of patients undergoing RA‐TKA and NG‐TKA.

	RA‐TKA	NG‐TKA	Significance
Hypertension diagnosis (%)	58.0	59.5	*p* < 0.0001
Dyslipidemia diagnosis (%)	44.3	47.3	*p* < 0.0001
Sleep apnoea diagnosis (%)	13.9	12.8	*p* < 0.0001
Chronic anaemia (%)	4.9	6.9	*p* < 0.0001
Alcohol abuse (%)	0.0	0.0	*p* = 0.308
Osteoporosis (%)	3.8	4.3	*p* < 0.0001
Mental disorders (%)	0.3	28.8	*p* < 0.0001
Parkinson's disease (%)	0.5	0.6	*p* = 0.532
Type 2 diabetes (%)	19.4	0.2	*p* < 0.0001
Renal disease (%)	5.6	6.8	*p* < 0.0001
CHF (%)	1.2	1.3	*p* = 0.004
Chronic lung disease (%)	0.1	5.5	*p* < 0.0001

Abbreviations: chf, chronic heart failure; NG‐TKA, navigation‐guided total knee arthroplasty; RA‐TKA, robotic‐assisted total knee arthroplasty.

Table 2 presents the prevalence of specific pre‐existing health conditions amongst patients undergoing RA‐TKA and NG‐TKA, highlighting statistically significant differences between the two groups.

As shown in Table 3 mortality rates during hospitalisation were minimal for both groups,

**Table 3:** Comparison of hospitalisation outcomes and costs between RA‐TKA and NG‐TKA.

	RA‐TKA (%)	NG‐TKA (%)	Significance
Died during hospitalisation	0.024	0.018	*p* = 0.342
Length of stay mean in days	1.89 ± 1.584	2.1 + 1.248	*p* < 0.0001
Total charges mean in $	66180 ± 39746	66251 ± 35879	*p* = 0.669

Abbreviations: NG‐TKA, navigation‐guided total knee arthroplasty; RA‐TKA, robotic‐assisted total knee arthroplasty.

A notable difference emerged in the mean LOS, where patients undergoing NG‐TKA had a slightly longer duration (2.1 days) compared to RA‐TKA (1.89 days).

The mean total charges did not show a significant difference between RA‐TKA and NG‐TKA.

Table 3 presents a comparative analysis of hospitalisation outcomes and costs between patients undergoing RA‐TKA and NG‐TKA, including in‐hospital mortality, LOS and total charges.

Table 4 provides a detailed overview of the prevalence of specific complications between RA‐TKA and NG‐TKA.

**Table 4:** Incidence of postoperative complications in RA‐TKA and NG‐TKA.

	RA‐TKA	NG‐TKA	Significance
Blood loss anaemia	11.671	14.185	*p* < 0.0001
Blood transfusion	0.598	1.240	*p* < 0.0001
Pulmonary oedema	0.0306	0.066	*p* < 0.0001
Venous thromboembolism	0.196	0.254	*p* = 0.006
Acute kidney injury	1.356	1.483	*p* = 0.016
Heart failure	0.098	0.112	*p* = 0.112
Pneumonia	0.086	0.118	*p* = 0.025
Pulmonary embolism	0.079	0.077	*p* = 0.861
Stroke	0.000	0.000	*p* = 1

Abbreviations: NG‐TKA, navigation‐guided total knee arthroplasty; RA‐TKA, robotic‐assisted total knee arthroplasty.

In terms of blood‐related complications, RA‐TKA patients demonstrated a lower incidence of blood loss anaemia. The rate of blood transfusion was also lower in the RA‐TKA group compared to the NG‐TKA group.

Pulmonary oedema displayed a statistically significant difference between the two groups, with a lower prevalence in RA‐TKA patients compared to NG‐TKA patients.

Heart failure, pulmonary embolism and stroke did not demonstrate statistically significant differences between the RA‐TKA and NG‐TKA groups.

Table 4 provides an overview of the prevalence of specific postoperative complications amongst patients undergoing RA‐TKA and NG‐TKA, highlighting statistically significant differences between the two groups.

In order to mitigate any potential bias arising from pre‐existing chronic anaemia, we utilised logistic regression modelling, incorporating chronic anaemia diagnosis as a control variable, to scrutinise the impact of the surgical system on postoperative outcomes.

When examining blood transfusion rates, postadjustment for chronic anaemia, patients undergoing navigation‐assisted surgery displayed an elevation in the odds of requiring blood transfusion compared to their robotic counterparts (odds ratio [OR] = 2.027, *p* < 0.001). Similarly, the logistic regression model investigating blood loss anaemia showed that patient in the navigation group exhibited 1.276 times higher odds of encountering blood loss anaemia relative to those in the robotic group (OR = 1.276, *p* < 0.001), even after adjusting for chronic anaemia.

When addressing potential biases tied to chronic kidney conditions, logistic regression modelling incorporated CKD diagnosis and RA‐TKA and NG‐TLA as covariates. Findings revealed that no difference in the odds for acute kidney injury (AKI) between RA‐TKA and NG‐TKA after adjusting for CKD.

## DISCUSSION

The most important finding of the present study was the significant decrease in complications and hospitalisation duration for RA‐TKA patients compared to NG‐TKA patients, without a significant difference in hospitalisation costs.

Our analysis of RA‐TKA and NG‐TKA procedures from 2016 to 2019 shows a substantial increase in RA‐TKA utilisation, rising from 11% in 2016 to 55% in 2019. This trend suggests growing recognition of the benefits of RA‐TKA.

RA‐TKA patients were discharged 0.21 days earlier than NG‐TKA patients. While this difference supports the feasibility of outpatient TKA, the clinical relevance may be more related to perioperative management than the surgical procedure itself. Our findings align with previous research indicating a reduction in LOS with RA‐TKA compared to conventional TKA [19].

RA‐TKA also demonstrated reduced postoperative blood loss anaemia, consistent with existing literature [1, 5, 9, 13, 14, 24]. The atraumatic approach of RA‐TKA, which minimises soft tissue damage, likely contributes to decreased intraoperative blood loss.

To address potential confounding factors such as pre‐existing chronic anaemia, we used logistic regression modelling. Even after adjusting for chronic anaemia, the association between RA‐TKA and reduced blood loss anaemia remained significant. This strengthens the validity of our findings and suggests improved patient recovery with RA‐TKA.

Robotic‐assisted surgery may contribute by potentially minimising blood loss through its precise movements and minimally invasive techniques [2, 3, 6, 13, 14, 22]. This could help maintain better kidney function and reduce AKI risk. Additionally, faster operative times and less tissue disruption associated with RA‐TKA may lead to quicker recovery and earlier mobilisation, potentially lowering the risk of deep vein thrombosis and pulmonary oedema. However, further research is needed to solidify these potential benefits.

Financially, there was no significant difference in mean total charges between RA‐TKA ($66,180) and NG‐TKA ($66,251) (*p* = 0.669). Both methods incurred higher costs than conventional TKA, but the potential benefits of RA‐TKA, such as reduced blood loss and shorter hospital stays, may lead to long‐term cost savings [27].

A previous study found differences in costs for robotics compared to navigation‐assisted surgeries, suggesting variability in financial outcomes [2]. However, our study did not show a significant difference in costs between RA‐TKA and NG‐TKA. This discrepancy may be attributed to the more recent data used in our study, which reflects advancements and optimisations in robotic‐assisted surgery over the years. Additionally, our study includes a much larger data set of RA‐TKA procedures, capturing the increasing utilisation of this technology and providing a more comprehensive analysis. The larger sample size and updated data enhance the reliability of our findings, suggesting that the financial impact of RA‐TKA has become more comparable to NG‐TKA as the technology has matured and become more widespread.

The reduced complication rate in RA‐TKA, without increased costs, supports its increased utilisation. This challenges the notion that medical innovation always leads to higher costs [6, 11, 27]. While the initial costs of RA‐TKA may be substantial, the long‐term economic benefits due to reduced complications and LOS can be significant, especially in high‐volume settings.

### Limitations

Our study has several limitations. First, we did not distinguish between different types of robotic assistance systems (image‐based vs. imageless, active vs. semiactive). Different systems may have varying outcomes, and this should be explored in future research. Second, the robotic group of patients had a higher socioeconomic level with better general health and fewer comorbidities, which could contribute to the lower rate of complications observed in this group. This difference may account for some of the benefits attributed to the robotic system. The NIS database, despite its comprehensive nature, may contain coding errors and lacks long‐term follow‐up data [12, 17]. Additionally, variations in surgeon preferences and patient selection could introduce bias. We addressed this by adjusting for comorbidities, showing that RA‐TKA still resulted in fewer complications.

Future studies should focus on the long‐term cost‐effectiveness and clinical outcomes of RA‐TKA versus NG‐TKA. Understanding these factors will help in making informed healthcare decisions that balance patient care improvements with financial sustainability [16].

Despite these limitations, our study has several strengths. It uses recent, comprehensive data from the largest all‐payer inpatient care database in the United States, providing a thorough analysis of demographics, health comorbidities and early postoperative outcomes. The focus on the early postoperative period offers insights that could help reduce future complications and improve long‐term outcomes. The use of ICD‐10 coding enhances the study's relevance and applicability [13].

## CONCLUSION

RA‐TKA reduces early postoperative complications and hospital LOS compared to NG‐TKA without increasing total costs. This technology offers safer surgical management for patients. Further studies on the short‐ and long‐term outcomes of RA‐TKA and NG‐TKA are needed to fully understand their potential.

## AUTHOR CONTRIBUTIONS

**David Maman**: Conducted major parts of the work; data analysis and manuscript writing. **Lior Laver**: Manuscript writing. **Roland Becker**: Provided clinical expertise. **Assil Mahamid**: Contributed to manuscript revisions. **Yaron Berkovich**: Conceived the study idea and mentored the project.

## CONFLICT OF INTEREST STATEMENT

The authors declare no conflict of interest.

## ETHICS STATEMENT

The study was conducted under exempt status granted by the institutional review board, and the requirement for informed consent was waived due to the deidentified nature of the NIS data set.

## Supporting information

Supporting information.

## Data Availability

The data that support the findings of this study are available from the National Inpatient Sample (NIS). However, restrictions apply to the availability of these data, which were used under license for the current study, and so are not publicly available. Data are available from the authors upon reasonable request and with permission of the NIS.
