# Supplementary material for: Robotic‐assisted total knee arthroplasty reduces postoperative complications and length of stay without increased cost compared to navigation‐guided techniques: A national analysis
Source: Knee Surg Sports Traumatol Arthrosc. 2024 Jul 2;33(1):336–42. doi: 10.1002/ksa.12348 (PMC11716347; doi:10.1002/ksa.12348)
Supplement: Supplementary file 1 — Supporting information. [file KSA-33-336-s001.docx]

| ICD 10 CODES / PROCEDURE CODE |  |
| --- | --- |
| 0SRC069, 0SRC06A, 0SRC06Z, 0SRC07Z, 0SRC0J9, 0SRC0JA, 0SRC0JZ, 0SRC0KZ, 0SRC0L9, 0SRC0LA, 0SRC0LZ, 0SRD069, 0SRD06A, 0SRD06Z, 0SRD07Z, 0SRD0J9, 0SRD0JA, 0SRD0JZ, 0SRD0KZ, 0SRD0L9, 0SRD0LA, 0SRD0LZ | Total knee arthroplasty procedure |
| 8E0Y0CZ,8E0YXCZ | Robotic Assisted Procedure of Lower Extremity |
| 8E0YXBZ, 8E0YXBG,8E0YXBF | Computer Assisted Procedure of Lower Extremity |
| I5021, I5031, I5033, I5041, I5043 | Heart Failure |
| N170, N171, N172, N178, N179 | Acute Kidney Injury |
| I2101, I2102, I2109, I211, I2119, I2111, I212, I2129, I213, I214, I219 | Acute Coronary Artery Disease |
| I60, I61, I62, I63, I650, I688, O873, O2250, O2251, O2252 | Stroke |
| J810, J811, I501 | Pulmonary Edema |
| I10(start with) | Hypertension |
| D62 (start with) | Blood Loss Anemia |
| J189, J159, J22 | Pneumonia |
| I2602, I2609, I2692, I2699 | Pulmonary Embolism |
| I82401, I82402, I82403, I82409, I82411, I82412, I82413, I82419, I82421, I82422, I82423, I82429 | DVT |
| E78(start with) | Dyslipidemia |
| G473 | Obstructive Sleep Apnea |
| D64(start with) | Chronic Anemia |
| F10 | Alcohol Abuse History |
| M81, M82 | Osteoporosis |
| F (start with) | Mental Disorders |
| G20 (start with) | Parkinson Disease |
| E11 (start with) | Type 2 Diabetes Mellitus |
| N18 (start with) | Chronic Kidney Disease |
| I500, I501, I509 | Congestive Heart Failure |
| J44 (start with) | Chronic Lung Disease |
|  |  |
